# Supplementary material for: Edible Coatings Containing Oregano Essential Oil Nanoemulsion for Improving Postharvest Quality and Shelf Life of Tomatoes
Source: Foods. 2020 Nov 4;9(11):1605. doi: 10.3390/foods9111605 (PMC7694186; doi:10.3390/foods9111605)
Supplement: Supplementary file 1 [file foods-09-01605-s001.pdf]

foods

MDPI

Supplementary material

Edible coatings containing oregano essential oil nanoemulsion for improving postharvest quality and shelf life of tomatoes

Annachiara Pirozzi [1], Vittoria Del Grosso [2], Giovanna Ferrari [1,2] and Francesco Donsì [1,*]

[1] Department of Industrial Engineering, University of Salerno, via Giovanni Paolo II, 132, 84084 Fisciano, SA, Italy; apirozzi@unisa.it, gferrari@unisa.it, fdonsi@unisa.it
[2] ProdAl Scarl, via Giovanni Paolo II, 132, 84084 Fisciano, SA, Italy; v.delgrosso@prodalricerche.it
* Correspondence: fdonsi@unisa.it; Tel: +39 089 964135

Received: date; Accepted: date; Published: date

1. Calibration curve of OEO

The nanoemulsions were tested in the Franz cells to calculate the effective diffusivity of OEO through a cellulose membrane. To evaluate the concentration of OEO in all analysed samples, the calibration curve (Figure S1) was prepared by reading the absorbance of the Folin-Ciocoltaeu reagent, at a wavelength of 765 nm, when mixed with serial dilutions of the nanoemulsion. The dilutions were selected on the basis of the expected concentration in the receptor compartment of the Franz cells.

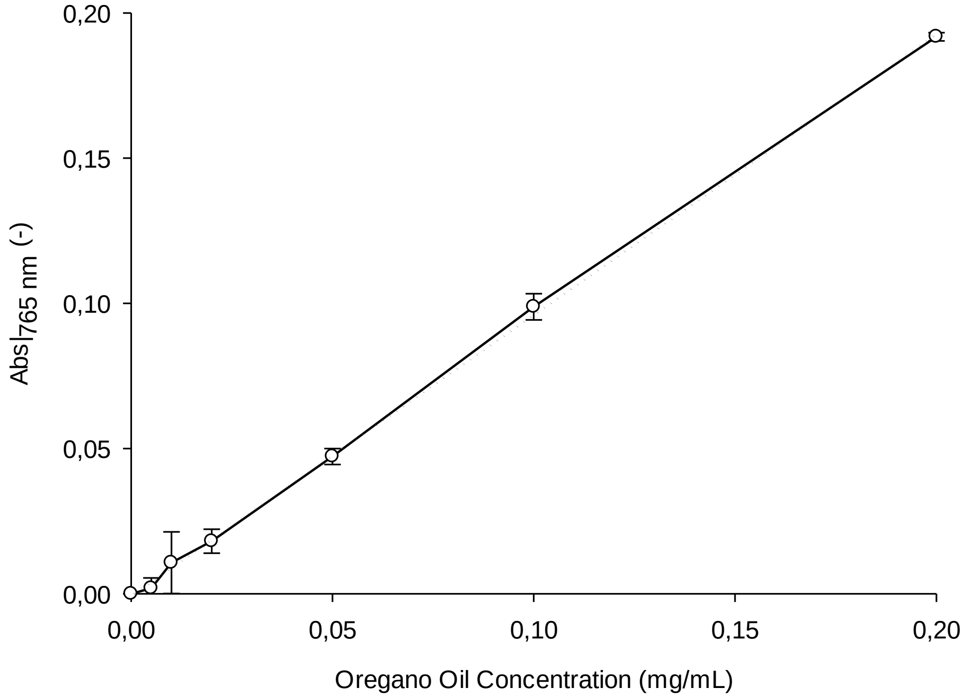

Figure S1. Calibration curve for UV-Vis absorbance of OEO.

The linear correlation between the UV-VIS absorbance and the OEO concentration was very high (R^2= 0.9993), and could be expressed through equation S1.

$$Abs|_{745\ nm} = 963.34 \cdot [OEO] \quad \text{(S1)}$$

Moreover, from calibration curve, according to Lambert Beer's law, it is possible to determine the molar absorptivity of OEO at 765 nm, which is about ε=A/(C·L)=963,34 mL/(g·cm).

2. Coating thickness optimization

The RSM was used to determine the number of experiments and to explore the effect of the three independent, i.e. dipping time (X_1) and concentrations of the coating and cross-linking solutions (X_2 and X_3), on the resulting response variable, the thickness of the coating layers (Y_1), listed in Table S1.

Table S1. The Central Composite design used, with the indication of the actual value and the coded level (in parenthesis) of the three variables and the response variable.

**Standard Order**	**Run Number**	**Dipping time (s) X_1**	**Concentration (% w/w)**		**Thickness (μm) Y_1**
			**Calcium chloride X_2**	**Sodium alginate X_3**	
**1**	1	10 (-1)	0.250 (-1)	0.250 (-1)	0*
**13**	2	155 (0)	3.625 (0)	0.250 (-1)	0*
**7**	3	10 (-1)	7.000 (+1)	5.000 (+1)	84,6
**10**	4	300 (+1)	3.625 (0)	2.625 (0)	35,7
**11**	5	155 (0)	0.250 (-1)	2.625 (0)	15,5
**18**	6	155 (0)	3.625 (0)	2.625 (0)	28,5
**14**	7	155 (0)	3.625 (0)	5.000 (+1)	54,7
**15**	8	155 (0)	3.625 (0)	2.625 (0)	48,6
**12**	9	155 (0)	7.000 (+1)	2.625 (0)	29,4
**5**	10	10 (-1)	0.250 (-1)	5.000 (+1)	11,5
**2**	11	300 (+1)	0.250 (-1)	0.250 (-1)	0*
**4**	12	300 (+1)	7.000 (+1)	0.250 (-1)	0*
**8**	13	300 (+1)	7.000 (+1)	5.000 (+1)	105,1
**19**	14	155 (0)	3.625 (0)	2.625 (0)	53,6
**3**	15	10 (-1)	7.000 (+1)	0.250 (-1)	0*
**17**	16	155 (0)	3.625 (0)	2.625 (0)	50,6
**16**	17	155 (0)	3.625 (0)	2.625 (0)	55,3
**20**	18	155 (0)	3.625 (0)	2.625 (0)	51,1
**9**	19	10 (-1)	3.625 (0)	2.625 (0)	22,1
**6**	20	300 (+1)	0.250 (-1)	5.000 (+1)	31,4

* Non-uniform coating observed on tomato surface.

The figure S2 illustrates the tomato samples coated according to the combination of the three independent variables of Table S1. This picture confirm the results obtained for the thickness of the coating layers. In fact as it's noticeable the layer obtained form run at lower concentrations is less visible for lower concentrations then for those at higher concentrations.

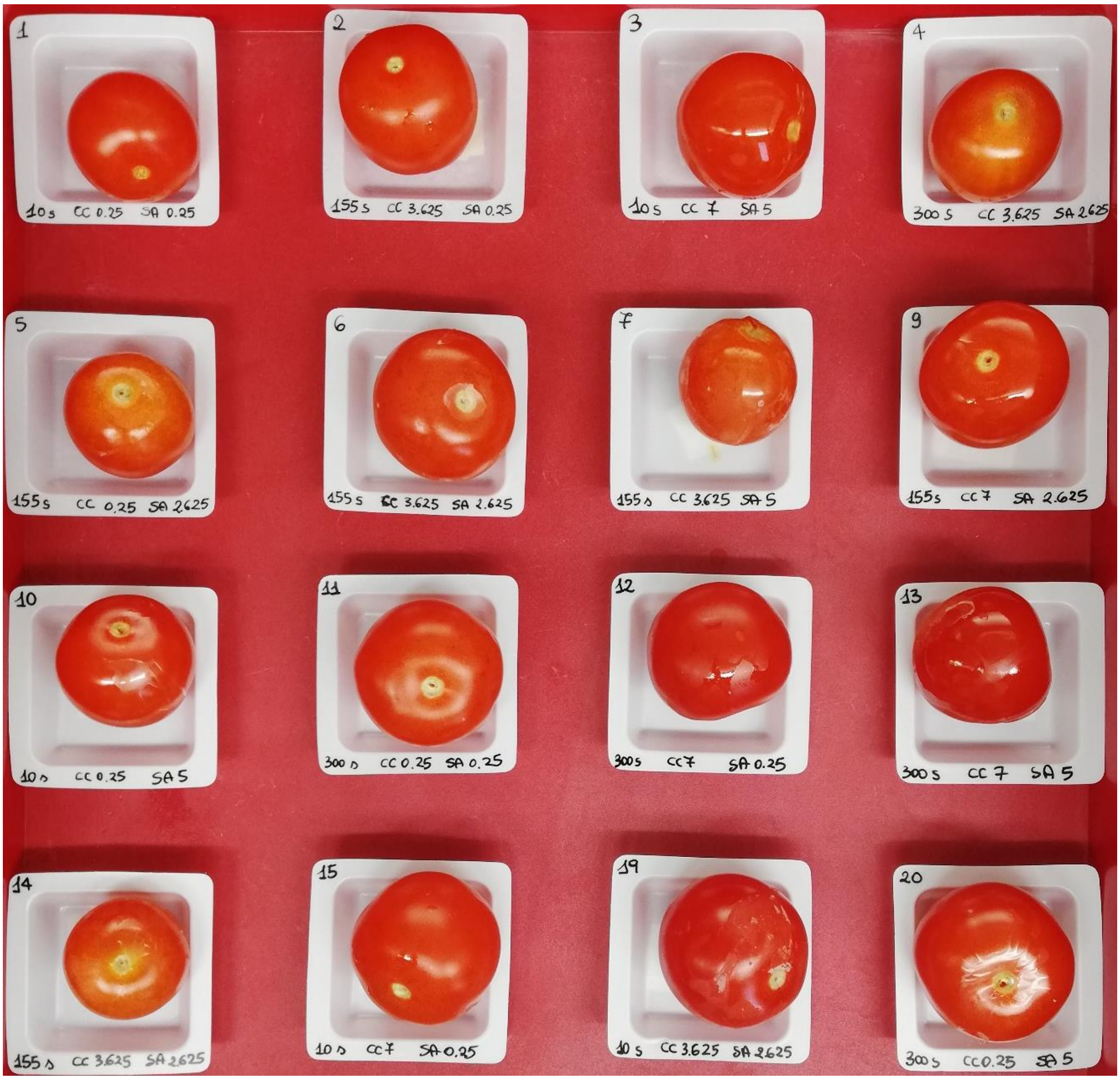

Figure S2. Picture of coated tomatoes at different concentrations of the coating and cross-linking solutions.

Details about regression analysis and ANOVA involved in the fitting model of film thickness response are shown in Table S2.

Table S2. Regression coefficients, R^2, F-test values (F_{reg}), and probability p-values for thickness response.

Source	Coefficient	Sum of squares	F_{reg}	p-value
β_0	0.0339	0.0003	12.31	0.0001
β_1	0.0054	0.0026	1.53	0.2373
β_2	0.0161	0.0083	13.60	0.0027
β_3	0.0287	4.88E-08	43.44	< 0.0001
β_{12}	0.0001	0.0002	0.0003	0.9875
β_{13}	0.0050	0.0027	1.07	0.3193
β_{23}	0.0184	0.0025	14.19	0.0024
ε		0.0025		
Lack of fit		0.0020	2.58	
Pure error		0.0005		
Total		0.0165		
R^2		0.8503		
Adjusted R^2		0.7812		
Predicted R^2		0.6564		
Stand. Dev.		0.0138		
Mean		0.0339		
PRESS		0.0057		
CV		0.4068		

The F-test value F_{reg} of the coefficients of the polynomial model (β_0) equal to 12.31 implies the model is significant. There is only a 0.01% chance that a F_{reg} this large could occur due to noise. The F-test value for lack of fit of 2.58 implies the lack of fit is not significant relative to the pure error. There is a 15.55% chance that an F-test value for lack of fit this large could occur due to noise. Finally, the values obtained for R^2 and $adj\text{-}R^2$ (0.8503 and 0.7812, respectively) suggest that the regression model was suitable to accurately describe the observed experimental data.

3. Contact angle of film forming solutions over time

Figure S3 shows the contact angle of water and film solutions, as a function of time elapsed from droplet deposition, on tomato skin, using alginate coating solutions without and with OEO nanoemulsion, in comparison with pure water. In all cases, the contact angle instantly reached an asymptotic value and substantially constant values were recorded over the 120-s experiment, with a reduction of only 1.5%, 2.4% and 4.8% for water, sodium alginate and sodium alginate with OEO, respectively, between the beginning and the end of the experiment.

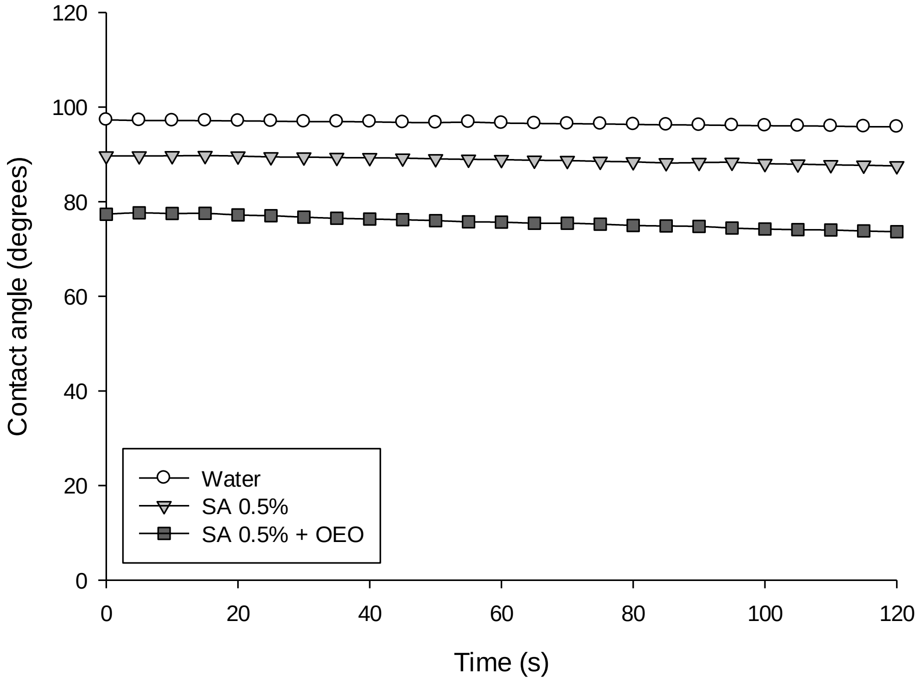

Figure S3. Contact angle over time of solutions on tomato skins (solution formulation given in the legend).

4. Color measurement

Tomato surface color was directly measured with a CR-400 Minolta chroma meter (Minolta, Inc., Tokyo, Japan). Color was measured using the CIE L*, a*, b* coordinates. The instrument was calibrated using a standard white reflector plate. Ten readings were made in each sample by changing the position of the tomato pieces.

The effect in the color parameters L*, a*, b* of tomatoes coated with calcium chloride cross-linked sodium alginate, with or without the addition of OEO nanoemulsion, has been evaluated (Table S3).

Table S3. Changes in the color parameters of tomato coated with calcium chloride cross-linked sodium alginate (with or without essential oils) during storage.

	Control	SA 0.5%	SA 0.5% + OEO
**L***			
0	39.54 ± 1.37^{b,A}	39.97 ± 2.26^{a,A}	40.43 ± 1.99^{b,A}
2	38.80 ± 1.26^{ab,A}	38.59 ± 1.51^{a,A}	38.64 ± 1.03^{ab,A}
3	38.28 ± 1.00^{ab,A}	38.92 ± 1.75 ^{a,A}	39.12 ± 1.38^{ab,A}
7	37.83 ± 0.79^{a,A}	38.87 ± 1.28^{a,A}	38.50 ± 1.32^{a,A}
10	37.92 ± 1.26^{a,A}	38.96 ± 1.26^{a,A}	38.82 ± 1.50^{ab,A}
15	37.23 ± 1.41^{a,A}	37.91 ± 1.60^{a,A}	38.37 ± 1.22^{a,A}
**a***			
0	29.77 ± 1.68^{a,A}	34.48 ± 1.92^{b,B}	36.40 ± 1.83^{b,B}
2	29.81 ± 2.59^{a,A}	34.37 ± 0.99^{b,B}	34.11 ± 1.82^{a,B}
3	28.36 ± 2.22^{a,A}	33.52 ± 1.67^{ab,B}	33.79 ± 1.44^{a,B}
7	27.90 ±1.76^{a,A}	32.23 ± 1.43^{ab,B}	32.46 ± 2.11^{a,B}
10	28.21 ± 2.44^{a,A}	32.49 ± 1.68^{ab,B}	32.68 ± 1.39^{a,B}
15	27.42 ± 2.49^{a,A}	31.37 ± 2.39^{a,B}	32.31 ± 1.61^{a,B}
**b***			
0	22.80 ± 2.23^{b,A}	24.31 ± 3.88^{b,AB}	27.06 ±3.36^{b,B}
2	21.76 ± 2.10^{ab,A}	23.27 ± 2.85^{ab,AB}	24.46 ± 1.39^{ab,B}
3	20.55 ± 1.58^{ab,A}	22.41 ± 2.86^{ab,AB}	23.74 ± 2.16^{a,B}
7	19.72 ±1.24^{b,A}	20.97 ± 2.14^{ab,AB}	22.25 ± 1.91^{a,B}
10	20.69 ± 2.79^{ab,A}	22.11 ± 2.08^{ab,A}	22.77 ± 2.61^{a,A}
15	19.67 ± 2.54^{a,A}	19.65 ± 2.80^{a,A}	22.18 ± 2.06^{a,A}

Data shown are the means (± standard deviation). Means for the same parameter in the same column (a, b) or in the same line (A–C) with different letters are significantly different ($p \leq 0.05$), according to ANOVA and the Tukeýs test.

L* values tended to decrease in all coated samples throughout the first 15 d of storage, but there is no significant ($p \leq 0.05$) difference in L* values between uncoated and coated samples for each storage day. The a* value is a measure of redness and is highly related to color changes of tomato. Edible coatings produced a modification of the a* coordinate compared to the control (Table S2), even if this difference was no visible. Regarding the yellowness values, a significant difference in b* values of samples coated with alginate containing OEO nanoemulsion in comparison with control, until 7 d of storage after which there is no more difference in b* value.
